# Transition of pediatric patients with an Auto-inflammatory Disease: an alternative version of the *Daedaulus and Icarus* myth

**DOI:** 10.31138/mjr.29.3.156

**Published:** 2018-09-27

**Authors:** Polyxeni Pratsidou-Gertsi

**Affiliations:** Pediatric Immunology and Rheumatology Referral Center, First Dept. of Pediatrics, Aristotle University, Hippokration Hospital, Thessaloniki, Greece

**Keywords:** Auto-inflammatory Disease, Transition, Transitional Care, Continuity of care, Adolescent health

## Abstract

Progress in the pediatric Auto-inflammatory Diseases (AIDs) has led to improved long-term outcome and the increased pool of pediatric patients who require lifelong monitoring. Implementation of a successful stepwise transition in patients with AIDs denotes the presence of a structured flexible and individualized policy that ensues the stepwise move from family-based pediatric care to adult patient one. This process aims to equip the young adult with self-management skills and the ability to enjoy life even under the burden of a chronic disease. Transition, thus, is a continuously evolutionary process that assists adolescents and young adults with an AID to move into a future that their predecessors with similar diseases never needed to experience. This review, using the myth of Daedalus and Icarus as a scaffold, presents the contemporary profile of the adolescent patient, comments on the evidence derived from Transition recommendations, and emphasizes the need of periodic quantitative assessments to assess the efficacy of the Transition plan. Upon the completion of the transfer to the Adult Center, monitoring of the patient’s active participation will support his/her engagement in the new setting.

“Icarus – Daedalus’ son – was excited for his ability to fly over the sea waves, supported by wax-glued wings. Thrilled and self-confident, he ignored his father’s recommendations and warnings and started to fly higher, close to the sun. The heat softened the wax, his wings fell apart and Icarus started dropping. His life coacher (and father) was unable to hold him… The floating feathers testified his drowning in the Icarean Sea.^1^”

## TRANSITION

Transition is interwoven with human life. Birth is the first transition: from the intrauterine to the extra-uterine milieu. At Adolescence, there is a move from childhood to adulthood. It is the physical and behavioral development starting in puberty and ending in early adulthood (20–24 years of age). Puberty describes the distinct period of sexual maturation that occurs during Adolescence.^2^

In Adolescence, apart from this striking physical and sexual evolution - principally attributed to the influence of hormones - young people gradually experience emotional upheavals and think that they are omniscient, fearless and infallible.^3,4^ Thus, some of them balance between risk-taking and pleasure-seeking behaviors. Alternatively, Adolescence enhances abstract reasoning and cognition, development of identity and of the principal skills to carry out adult relationships and roles. Namely, this critical period calls for decision-taking regarding home residence, education/occupation, community integration, social and financial independence.^3,5^ This evolution, however, in the presence of any bystanding stress factor, is vulnerable to the development of psychological disorders, such as anxiety and depression.^6^ Thus, transition to Adulthood is a developmental challenge for all adolescents irrespective of their health status and for their parents, educators and health providers as well.^5,7–10^

### Transition and transitional care in Pediatric chronic Diseases

Transitional care, or Transition, in respect to health issues, denotes “the purposeful, planned movement of adolescents and young adults with chronic physical and medical conditions from child-centered to adult-oriented health care centers”.^5,8,9,11–13^ Thus, for adolescents with any chronic disease, such as Auto-inflammatory Diseases (AIDs), Transition is a double and demanding challenge.^7,11^ All these young people, besides this gradual bio-psychosocial evolution, need to receive training and support in order to develop skills for communication and the health-related self-management.^7,10–16^

Previous Greek publications on the Transition of Pediatric Rheumatic Diseases have highlighted the principles of an artful and successful Transition and have provided evidence for transitioning Juvenile Idiopathic Arthritis and pediatric SLE patients.^17–19^

### Transition in Pediatric Auto-inflammatory Diseases

Auto-inflammatory diseases (AIDs) are rare diseases characterized by the presence of chronic or recurrent systemic inflammation due to aberrant activation of innate immunity mediators and cells.^20–22^

In Greece, the majority of AIDs are classified among the Periodic Fever Syndromes and are mainly diagnosed in childhood. They include the Syndrome of Periodic Fever, Aphthous Stomatitis, Pharyngitis, Cervical Adenitis (PFAPA), Familial Mediterranean Fever (FMF), Hyper IgD Syndrome (HIDS) or Mevalonate Kinase Deficiency (MVK), and the Tumor necrosis factor Receptor-Associated Periodic Syndrome (TRAPS). All but PFAPA continue to adulthood.^21^

Advances in the pediatric AIDs in terms of earlier recognition, multi-system evaluation, targeted treatment and compliance predispose to an improved long-term outcome. Hence, there is a growing population of young people with AIDs who are candidates for Transition and “navigation” from the pediatric and family-focused health care to the adult one. ^23,24^

The latest relevant publications or web-sites including the EULAR/Paediatric Rheumatology European Society (PReS) Transition recommendations, provide the general frames for Transition in Pediatric Rheumatic Diseases.^24–26^ A search for AIDs Transition Recommendations found patient-support websites that offer links to General Recommendations on Transition written by the American College of Physicians, the American College of Rheumatologists or University Settings.^23^ All publications on Transition emphasize the necessity of a written transition policy for each Setting, in order to uneventfully transition and engage the patient in the adult care. ^5,7,9,13,14^

These policies should, however, take into consideration the local infrastructure and the family/patient cultural background.^27^ It is noteworthy that caregivers of Mediterranean countries differ from Northern European countries.^28^ They have the tendency to protect their “affected” offspring particularly during Adolescence, the vulnerable period he/she struggles for the educational transition and try to delay the “pruning” of the emotional family bonding.^27,28^ Our Transition Team in Hippokration Hospital (**Table 1**), occasionally witness the request of parent’s presence in the post-transitional appointments for the first follow-ups.

**Table 1.** Transition policy addressed to adults with Pediatric Rheumatic Diseases in Hippokration Hospital

**“Health-related” Stakeholders:** Pediatric Immunology and Rheumatology Referral Center, 1^st^ Dept. of Pediatrics, Aristotle University Thessaloniki (AUTH) Rheumatology Unit, 4^th^ Dept. of Internal Medicine, AUTH Patients and Parents **Transition team:** Pediatric Rheumatologists, Adult Rheumatologists, Liaison (Physiotherapist) **Model of transition:** Gradual patient education and comb evaluations in the Adult Outpatient Clinic post-transitional, for 2–3 follow-up appointments

Thus, the aim of the present narrative and systemic review is to review the literature and propose a tailored Transition policy for young Mediterranean people with an AID. An alternative version of the Daedalus and Icarus myth will be the scaffold of this simulation attempt by questioning whether Icarus’ flight failure was entirely his own error, attributed to disobedience.^1,29^

### Preparing the Transition

“Icarus fears the flight to an unknown terra…”

Young people are often hesitant to any transfer to unknown adult health settings. Yet, health-related Transition is not synonymous with a hand-off to adult health providers.^4,13,15,16,24,26^ This information should be continually provided to the family and patient, starting from early appointments with the presentation of the Transition plan to the family (**Table 2**). Transfer is thus the final moment and will be supported by his/her Transition team. ^9,12,13,25^

**Table 2.** Domains of the stepwise transition training

**Disease knowledge and cognition of:** Symptoms and signs, disease course and outcome The significance of disease logging using AIDAI and of the holistic management
**Progress regarding Shared Decision Making (SDM):** AIDAI contribution and self-management of treatment and FUs prerequisites to the SDM **Treatment:** Recording the improved self-compliance to medications and shrinking the parental “intrusion” **Appointments:** Preparation of the folder with the necessary documents, self-reporting the visits under a creative communication with the health providers in the absence of parents, self-booking future appointments and handling bureaucratic health-issues
**Self-tracking the progress in Transition** Sequential self-reports in pre-formatted questionnaires regarding readiness (TRAQ) and Service Satisfaction Development of realistic life expectations for the co-living with the AID Active contribution to the tailored transition

### Implementation

The implementation of the transition process in patients with AIDs involves several steps, described below. A coordinated cooperative plan is operated by the stakeholders; namely the transition team, the family and the patient. The recommendation underscores the mutual special handling of the transition, and the gradual multi-task education.^5,25–26^

#### Daedalus prepares the flight plan, as guided by the Goddess of wisdom, Athena

EULAR/PReS recommendations developed by experts, can be a flight compass.^25,29^ The involved health providers, under the coordination of the most experienced one, will form the Transition Team. The physician coordinator should also be in charge of managing the calendar of visits. The Team, skillful in managing adolescents and AIDs, will follow the steps of their designed transition policy; however, they will also be capable of modifying it, according to the family/patient progress and disease course. Without a tailored Transition plan, a young person may have a poor outcome and an impaired Health-Related Quality of Life, with a raised chance of non-compliance to periodic assessments in the new terra or malpractice of his/her self- care.^7,25,30,31^

Regardless of the type of disease, the key preparation is the gradual familiarity with the disease profile and management, escorted by a blooming trusted communication with the stakeholders.^25,26,32^

#### Icarus’ wings

Icarus’ contemporary flight wings are the growing skills for communication, decision making, and self-health management, aiming towards a healthy life-style and realization of the life-long co-existence with the AID.^25,32,33^ Compliance to medication – even if parenterally administered – is of high importance to “pilot” the safe flight.^34^

#### When is the ideal onset time for the “flight preparation”?

It starts with the announcement of diagnosis for the parents and for the patient in Adolescence.^25^ Of course, physical growth and developmental bio-psychosocial process do not run in parallel. The adolescent brain domains regarding feelings, community skills, impulses, critical thinking, and self-independence do not develop with the same rate, and may delay in young people that carry the burden of a chronic disease. Additionally, the endocrine system cross-talks with the maturing brain; and usually, females have a higher rate of evolution.^3,6,9,12,35^

Following diagnosis, the honest and clear-cut physician’s periodic updating of the family/patient regarding distinctive AID clinical features, mode of inheritance, disease course, Quality of Life and future expectations will result in compliance to guidelines and treatment. Reassuring of careful monitoring under medication in terms of family planning will be provided upon request.^25,36^ Furthermore, documented information regarding the non-compliance consequences may motivate the adolescent to take responsibility and participate consciously in the Shared Decision-Making process. The importance of the Transition, as expected, will be gradually realized by the patient/family. They will be educated on the need for future periodic assessments by an adult skilled Rheumatologist, who has the experience to manage adult patient problems.^16,26,30,37^

#### Education on Piloting

The trusted interactive discussion among the stakeholders under the law of confidentiality targets empowerment through the individualized preparation plan and cognition of the disease. Overtime, parents need to understand their “shrinking role” in terms of their off-spring’s advocacy and leave him/her more space; ideally letting the adolescent alone with the Transition team.^38^ Indeed, some adolescents under parental presence may fear that they will be viewed negatively and may withhold information from the Team. Even in parental absence, confidentiality for them may be difficult if they suspect that the information provided will be forwarded to parents.^6,15,16,25,26,38^

Another important aspect is the longer time that should be devoted to the adolescent patient as compared to adults or children, despite over-crowded settings. It is critical for his/her transition preparation, as quick reference can create anxiety and mistrust.^8,38^

The Pediatric Transition Team identifies and records the patient/family capability to respond to the circumstances and overcome probable obstacles by sequential assessments with relative Questionnaires, evaluating Health e-literacy, Readiness and Service Satisfaction.^30,39,40^ The duration and rate of training thus depends not only on the patient’s age at diagnosis and the family’s cultural perceptions, but to his/her training Progress, as well. ^10,14,25,26,39,41^

Parents and the family pediatrician/physician will also conduct a continuous training at home, at school and in any extra-curriculum activity (**Table 3**). All educators involved should be familiar and periodically updated for the patient’s co-living with the disease as they constitute his/her Life coachers.^7,14^

**Table 3.** The family-life experientially educates the patient on the AID self-management

Parents co-organize with the transition team the AID education of the patient and the family members at home Parents co-organize with the transition team the AID education of the educational and extra-curriculum staff Parents support the recording of raising queries or discrepancies in the appointments, gradually co-recorded by parent/patient Membership and involvement in relevant Parents/Patients’ Associations Life adaptation of ALL family members in respect to the needs of the AID regarding family activities and obligations

### Avoiding barriers of a successful Transition (The waves)

During late Adolescence and onward, the brain limbic system that governs emotions, learning and critical thinking, cooperates with the frontal lobe for the optimal living adaptation and competence achievement. Empowering the patient/family though interactive conversation, and simulation-based training will effectively support the young patient in problem-solving; merely providing answers to prepared questions or suggesting ready solutions through the physician disease glance will not support increased retention of knowledge and skills.^3,4,15,25,29,33^

Barriers to good compliance can be attributed to the lack or misunderstanding of the AID nature and disease prioritizations, any adverse events, the AID impact in socializing or relationships, as well as parenteral administration of biologics. Poor compliance to these agents may not just be fear of needles, but reluctance of difficulties with self-administration, that my lead to social isolation.^25,31,33,34^

#### Daedalus promotes self-education

The patient’s recognition of the triggering episodic crises and any experience of non-drug compliance will experientially help him/her realize the consequences of disobedience. Typical triggers, such as heat and overuse for FMF or cold exposure to cryopyrin syndromes need to be emphasized since diagnosis and may stimulate the patient to avoid or properly manage them. Importantly, as self-e-literacy is a popular trend of adolescents, it can be an advantageous educational tool.^30,40^ Suggestion and demonstration of reliable web sites or forums, will awake the patient’s response to the context and validity over consecutive visits. In parallel, warning for the dangers of Web-fishing will be periodically highlighted.^40–41^

#### Daedalus uses wax to stick the wings

Suggested tools for the transition flight can be:

A. A portable paper or digital organizer

This can record the ongoing reports regarding the AID course and treatment and will facilitate unprovoked periodic outpatient visits. It may also include contact information of his/her health providers, or closest family/friends in case of emergency, the required laboratory or other periodic exam schedule, will help develop his self-care and monthly digital recording of any symptoms/signs during the disease course.^14,19,30^ The recent pre-formatted, meticulous but easy to fill in diary, the AIDAI (Auto-inflammatory Diseases Activity Index) may increase his awareness targeting to the disease taming.^42^

B. Virtual transition tours

Some institutions suggest simulation targeting to ease the transition propose web-based virtual tours to the new health care setting. They additionally upload interviews and staff presentation during their work procedure in order to alleviate the patients’ worries for the final transfer.^24,25^

C. Other self-assessment questionnaires

A valuable tool evaluating the improved holistic Transition Readiness since the commencement of training can be any preformatted questionnaire, such as the TRAQ (Transition Readiness Assessment Questionnaire) available online.^35,43^ The TRAQ evaluates the progress in the key domains of co-living with chronic disease: Medications management, appointment Keeping, tracking Health Issues, talking with Providers and managing daily activities. Parents are invited to fill in such questionnaires as well. In case of unraveled difficulties, individualized interactive training will be performed in the missing targets and the transition plan respectively modified or the final transfer delayed.^25,31,33,37^

Moreover, recording questionnaires by the adolescent’s response and aiming to evaluate his/her Satisfaction of the applied Health Service may support adherence to the Outpatient Clinic and promote his/her unprovoked preparation.^33,34,44,45^

#### Daedalus and Icarus fly in parallel over the life waves and not too close to the inflammatory beams

Upon verification of the patient’s (and family’s) global Transition readiness and absence of any disease flare or other physical or emotional stress, the final and orchestrated transfer by the Transition team will be conducted.^16,25,26,35^ To our experience, a couple of combined post-transitional assessments in association with the presence of the Liaison, will soothe the young person’s adaptation and maintenance in the new setting.

#### Icarus flight Health Passport

This is a pre-formatted document to communicate the AID profile to the Adult Health providers. This “Health Passport” is not just a discharge report; it is a detailed medical report capturing all aspects of the AID, and the patient’s overtime actions will be ideally sent prior to their first meeting with the young person (**Table 4**).^15,17,25,26^

**Table 4.** Medical report at the final stage of Transition for a patient with AID

Classification or diagnostic criteria of the Autoinflammatory Disease (AID) Genetic analysis (mutations/polymorphisms)-contribution to diagnosis establishment Relevant geo-epidemiology and family history supportive for the diagnosis Disease onset phenotype and work up Initial diagnosis and lag time to diagnosis Disease course including periodic activity scores Therapeutic management and response to treatment Medication tolerance and drug toxicity Damage development Revision of diagnosis Transition plan and progress (milestones achieved up to the transfer) Compliance over the disease course Comorbidities/complications (atherosclerosis, obesity, etc.) Vaccinations Medical reports of other specialties periodic eye/auditory assessment Academic performance Current academic/occupational and psycho-social status

#### Icarus safely lands on the new terra. What’s next?

The final uneventful transfer to the Adult Care does not preclude maintenance in the new Setting. The Liaison will definitely keep an eye on him/her. Efforts on gaining the young persons’ trust in the adult Care, continuous review of their Patient Reported Outcome Questionnaires on Service Satisfaction and Disease monitoring, the fruitful communication with the Liaison between the visits as well as his/her participation in relevant Associations or Alliances will further contribute to self-health management. Aftermath, “residual” visits of the pediatric Rheumatologists to the Adult Setting are welcome to assess their applied policy and secure the young persons that he/she has not been “abandoned”.^15,30,32,33,37,38,44^

## CONCLUSION

Transition is a continuously evolutionary process to help adolescents and young adults with AID move into a future that their predecessors with the same disease never needed to experience.^14,23^

During Transition unexpected barriers may emerge, but can be overcome by the flexibility of the Transition plan. Transition will allow young people to become autonomous, live in harmony with their AID and “land” on an appropriate patient-centered Care. Their transition will thus be another “success” story of Medicine.^14^ Any physician can be a Daedalus, since the Ancient Greek verb “δαιδάλω (Daidállō)” means “work artfully”.^1^
